# The stimulatory effect of fusobacteria on dendritic cells under aerobic or anaerobic conditions

**DOI:** 10.1038/s41598-022-14934-z

**Published:** 2022-06-23

**Authors:** Shigeo Koido, Sankichi Horiuchi, Shin Kan, Tsuuse Bito, Zensho Ito, Kan Uchiyama, Masayuki Saruta, Nobuhiro Sato, Toshifumi Ohkusa

**Affiliations:** 1 Division of Gastroenterology and Hepatology, Department of Internal Medicine, The Jikei University School of Medicine, Kashiwa Hospital, 163-1 Kashiwashita, Kashiwa City, Chiba, 277-8567 Japan; 2 Institute of Clinical Medicine and Research, The Jikei University School of Medicine, Kashiwa City, Chiba, Japan; 3 Division of Gastroenterology and Hepatology, Department of Internal Medicine, The Jikei University School of Medicine, Tokyo, Japan; 4 Department of Microbiota Research, Juntendo University Graduate School of Medicine, Tokyo, Japan

**Keywords:** Microbiology, Immunology

## Abstract

Fusobacteria have been suspected to be pathobionts of colon cancer and inflammatory bowel disease. However, the immunomodulatory properties that affect these inflammatory reactions in dendritic cells (DCs) under anaerobic and aerobic conditions have not yet been characterized. We directly assessed the stimulatory effects of anaerobic commensal bacteria, including fusobacteria, on a human DC line through coculture under aerobic or anaerobic conditions. Under aerobic or anaerobic conditions, stimulation of the DC line with all live commensal bacteria examined, except the probiotic *Lactobacillus delbrueckii subsp. bulgaricus* (*L. bulgaricus*), significantly increased the geometric mean fluorescent intensity (MFI) of marker proteins (HLA-ABC, HLA-DR, CD80, CD86, CD83, or CCR7) on the DC surface. In particular, both *Fusobacterium nucleatum* (*F. nucleatum*) and *Escherichia coli* (*E. coli*) significantly increased the expression of DC-associated molecules, except for CD83 under both aerobic and anaerobic conditions. The DC line stimulated with *Fusobacterium varium* (*F. varium*) significantly increased only CD80, HLA-ABC, and HLA-DR expression under anaerobic conditions. Moreover, differences in the levels of proinflammatory cytokines, such as IL-6, IL-8, and TNF-α, were detected in the DC line stimulated by all live commensal bacteria under either aerobic or anaerobic conditions. Under aerobic conditions, the DC line stimulated with *E. coli* produced significantly more IL-6, IL-8, and TNF-α than did the cells stimulated with any of the bacteria examined. When *E. coli* were used to stimulate the DC line under anaerobic conditions, TNF-α was predominantly produced compared to stimulation with any other bacteria. Compared to the DC line stimulated with any other bacteria, the cells stimulated with *F. nucleatum* showed significantly increased production of IL-6, IL-8 and TNF-α only under anaerobic conditions. In particular, *E. coli*, *F. nucleatum,* and *F. varium* strongly stimulated the DC line, resulting in significantly increased expression of surface molecules associated with DCs and production of inflammatory cytokines.

## Introduction

Mucosal surfaces in the intestinal tract are continuously exposed to both potential pathogens and beneficial commensal bacteria^1^. Commensal bacteria are normally considered tolerant to epithelial cells and immune cells^2,3^. However, these bacteria may become pathogenic and contribute to irritable bowel syndrome (IBS), inflammatory bowel diseases (IBDs) such as ulcerative colitis (UC) and Crohn’s disease (CD), and colorectal cancer (CRC)^4^. These symptoms depend on an intestinal immune homeostatic balance between tolerance and immunity that represents a unique regulatory challenge to the mucosal immune system^2^. Tolerance and activation of the human immune system are complex processes and depend on the amount and diversity of the microbiota at mucosal sites, where epithelial cells and antigen presenting cells (APCs), such as dendritic cells (DCs) and macrophages, play an important role^4,5^. Clearly, dysregulation of the gut microbiota affects both local and systemic compartments and is associated with several types of disease, such as autoimmunity, allergies and cancer^4^. The gastrointestinal tract is the primary site for the interaction between the gut microbiota (commensal and pathogenic) and the human immune system^5^. We have reported that certain commensal bacteria invade colonic epithelial cells, activating early intracellular signaling pathways to trigger host inflammatory reactions^6^. Understanding how the immune system is stimulated by the microbiota is of interest.

DCs are specialized APCs that play an important role in the immune system^7^. Their main function is to present antigens to T cells, activate primary T-cell responses, and determine whether these responses to commensal bacteria are immunogenic or tolerogenic^8,9^. DCs are also considered the main coordinators of both mucosal and systemic immune responses^10^. Therefore, the adaptive immune system may be influenced by disturbances in bacterial microbiota colonizing the gut, resulting in IBS, IBD, and CRC. We have previously reported that some commensal bacteria augment corticotropin-releasing factor (CRF) production by DCs, which may alter gut motor function and visceral perception prior to the development of IBS^11^. Moreover, our previous report also documented that intestinal bacteria, such as *Fusobacterium varium* (*F. varium*), contribute to the clinical activity of UC^12^. Therefore, individual members of commensal bacteria may have different abilities to activate immune responses through DCs. However, researchers have not yet determined how primary human DCs drive the polarization of helper T (Th) cells and regulatory T cells (Tregs) in the presence of commensal bacteria harboring unique immunomodulatory properties.

Moreover, DCs express various pattern-recognition receptors, including Toll-like receptors (TLRs), involved in recognizing a variety of microbial products^13,14^. The DC line used in this study expresses TLRs (TLR1, TLR2, TLR4, TLR7 and TLR9) and is capable of antigen capture, processing and presentation to T cells^15–17^. The endotoxin lipopolysaccharide (LPS), a cell wall component of gram-negative bacteria, induces an inflammatory response through TLR4 in the DC line^16,18^. Our previous report also indicated that the production of both interleukin (IL)-12p70 and IL-10 by the DC line occurs upon stimulation with LPS^19,20^. The surface phenotype and proinflammatory cytokines produced in the DC line were similar to those of monocyte-derived myeloid DCs (MoDCs), indicating that the DC line possesses characteristics of MoDCs^19,20^. Therefore, we used the DC line, which efficiently triggers immune responses, to assess the potential capacity of individual bacterial species to activate immune responses through the DC line.

Recently, *Escherichia coli* (*E. coli*) and fusobacteria have been suspected to be pathobionts of CRC and IBD. Specifically, *Fusobacterium nucleatum* (*F. nucleatum*) has been proposed to be a pathobiont of CRC^21,22^ and IBD^23^. We also reported that *F. varium* is associated with UC^24^ and is at least partially involved in the pathogenesis of colorectal adenoma and intramucosal CRC^25^. Moreover, CRC in southern Chinese populations is linked to *F. varium* as well as other fusobacteria, such as *F. nucleatum*^26^. Therefore, *Fusobacterium* species, such as *F. varium* and *F. nucleatum*, may be one of the more stimulatory bacteria influencing many immune cell populations, such as DCs, in the colon^27^.

Most previous studies with anaerobic bacteria have been conducted under aerobic conditions, which are not good conditions for the survival and function of anaerobic bacteria. We used anaerobic commensal bacteria isolated from the human intestine, namely, *F. varium*, *F. nucleatum*, *Bacteroides vulgatus* (*B. vulgatus*), *Clostridium clostridioforme* (*C. clostridioforme*), and *E. coli*, and examined the effect of bacterial flora in the gut on DCs under anaerobic conditions similar to intestinal environments. First, we developed an in vitro experimental model in which DCs and anaerobic bacteria react under anaerobic conditions. The stimulatory effects of these anaerobic bacteria on the DC line were directly assessed by establishing a coculture system with live bacteria under aerobic and anaerobic conditions. Here, we report that the DC line is able to directly mediate robust immune responses upon stimulation with commensal bacteria such as *E. coli*, *F. nucleatum*, and *F. varium* under anaerobic conditions, while the probiotic *Lactobacillus delbrueckii subsp. bulgaricus* (*L. bulgaricus*) limits this effect.

## Results

### Viability of the DC line stimulated with live commensal bacteria under aerobic or anaerobic conditions

**Figure 1:**
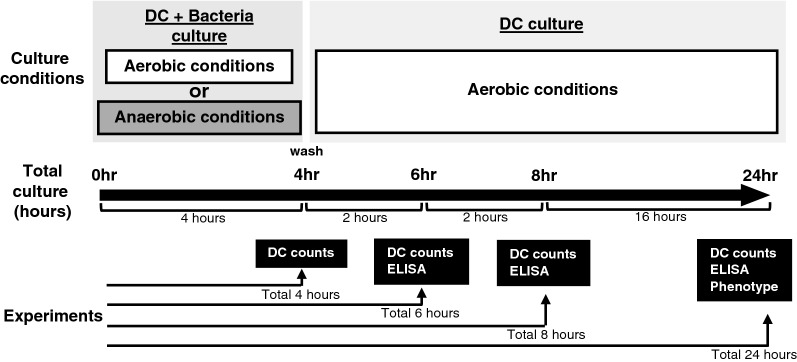
Stimulation of the DC line with live commensal bacteria. The human DC line was cocultured with each live commensal bacteria for 4 h under aerobic (white square) or anaerobic (black square) conditions. After coincubation, the DC line was washed to remove commensal bacteria and further incubated for 2, 4, or 20 h under aerobic (white square ) conditions (total of 6, 8, or 24 h).

**Figure 2:**
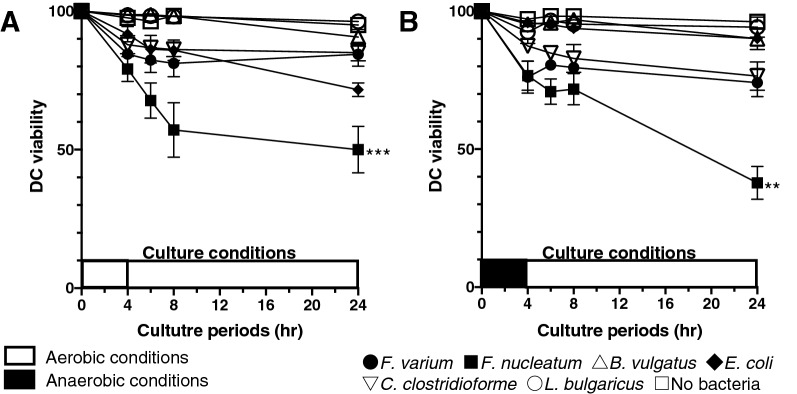
The viability of the DC line after coculture with live commensal bacteria under aerobic or anaerobic conditions. The DC line was cocultured with each live commensal bacteria for 4 h under aerobic (**A**; white square ) or anaerobic (**B**; black square ) conditions. After coincubation, the DC line was washed to remove commensal bacteria and further incubated for 2, 4, or 20 h under aerobic (white square ) conditions (total of 6, 8, or 24 h). Then, the percentage of viable cells was assessed at various culture periods. The results are presented as the means ± SD from three independent experiments. ** *p* < 0.01 and *** *p* < 0.001 compared to the control unstimulated DC line.

An analysis of DC viability in this experimental setting is important to assess the function and phenotype of the DC line stimulated with live commensal bacteria. The commensal bacteria used in this study are anaerobic (*F. varium*, *F. nucleatum*, *B. vulgatus*, and *C. clostridioforme*) or facultative anaerobes (*E. coli* and *L. bulgaricus*). After 4 h of coculture of the DC line with live commensal bacteria without antibiotics under aerobic or anaerobic conditions, DCs were washed and then cultured with antibiotics under aerobic conditions for an additional 0, 2, 4, and 20 h (total of 4, 6, 8, and 24 h, respectively) (Fig. 1). According to Dunnett’s multiple comparisons test, no significant difference in viability was observed between the unstimulated DC line and the DC line stimulated with certain commensal bacteria (*F. varium*, *B. vulgatus*, *C. clostridioforme*, *E. coli*, or *L. bulgaricus*) under aerobic or anaerobic conditions for a total of 24 h. However, compared to the unstimulated DC line, the viability of DC lines cocultured with *F. nucleatum* was significantly decreased after 24 h under either aerobic (*p* = 0.0006) or anaerobic (*p* = 0.0015) conditions (Fig. 2). The viability of the stimulated DC line was not significantly different from that of the unstimulated DC line until 4 h of culture with any of the live bacteria (Fig. 2).

### The change in the commensal bacterial number after culture under aerobic or anaerobic conditions in complete medium

As the commensal bacteria used in this study are anaerobic or facultative anaerobes, aerobic culture conditions may be disadvantageous for anaerobic bacteria survival and result in bacterial death^28^. Therefore, the effects of culture on these bacteria under aerobic or anaerobic conditions were also assessed. The changes in viable bacterial counts were evaluated logarithmically as previously reported^28^. Four hours of culture of certain viable bacteria (*F. varium*, *F. nucleatum*, *B. vulgatus*, *C. clostridioforme*, and *L. bulgaricus*) under either aerobic or anaerobic conditions did not induce significant bacterial death (Table 1). However, only the number of *E. coli* increased approximately 10 times or more during 4 h of culture under aerobic or anaerobic conditions (Table 1).

**Table 1:** The percent change of live commensal bacterial count (log 10 cgf/mL) after 4 h incubation under aerobic or anaerobic conditions.

	Aerobic condition	Anaerobic condition
*F. varium*	102.07 ± 1.00	102.55 ± 1.15
*F. nucleatum*	96.32 ± 0.51	99.66 ± 1.40
*B vulgatus*	101.00 ± 0.88	104.2 ± 1.83
*C. clostridioforme*	102.07 ± 0.13	101.86 ± 0.57
*E. coli*	116.84 ± 6.38	115.94 ± 4.73
*L. bulgaricus*	99.62 ± 2.39	96.06 ± 2.73

### Phenotypic characterization of the DC line stimulated with live commensal bacteria under aerobic or anaerobic conditions

**Figure 3:**
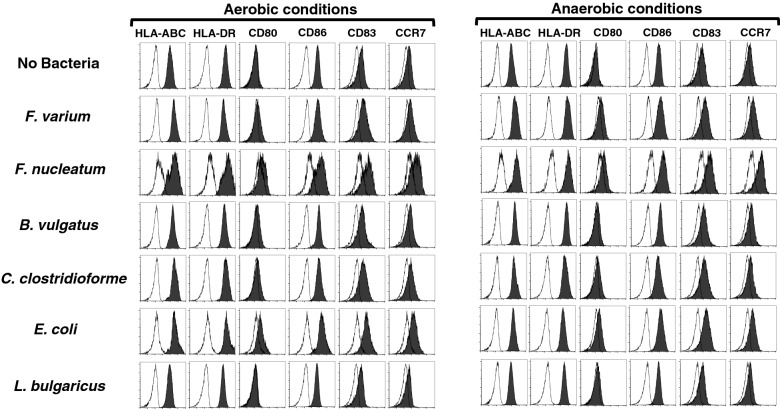
Phenotypic characterization of the DC line stimulated with live commensal bacteria under aerobic (left panel) or anaerobic (right panel) conditions for 4 h followed by an additional 20 h under aerobic culture conditions. Expression of the indicated antigens (black) or unstained controls (white) on the DC line was analyzed using flow cytometry. Similar results were obtained in three independent experiments.

**Figure 4:**
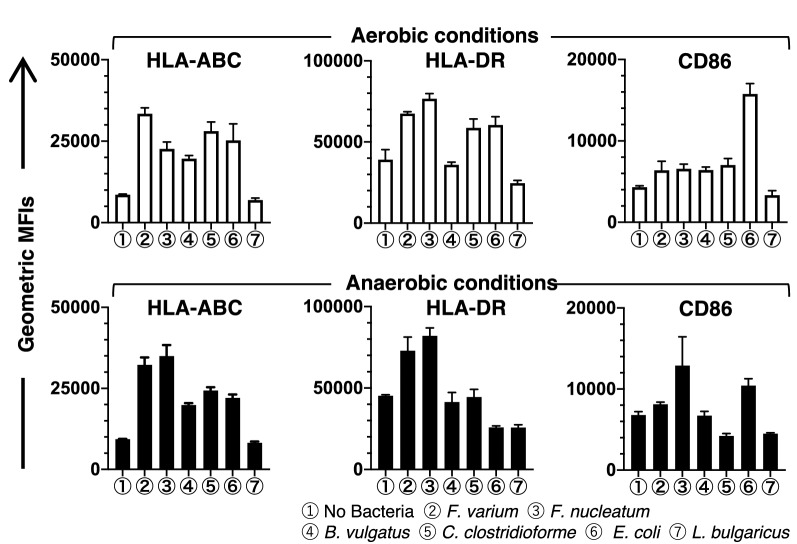
The geometric MFI of the indicated molecules (HLA-ABC, HLA-DR, or CD86) on the surface of the DC line stimulated with live commensal bacteria under aerobic (upper panel) or anaerobic (lower panel) conditions for 4 h followed by an additional 20 h of culture under aerobic conditions (total 24 h). The geometric MFI indicated the expression levels of the indicated molecules on the commensal bacteria-stimulated DC line subtracted from those on the unstimulated DC line incubated without mAbs. The results are presented as the means ± SD from three independent experiments.

DCs were cocultured with or without each live commensal bacterial strain for 4 h under aerobic or anaerobic conditions to analyze whether live commensal bacteria induce phenotypic maturation of the DC line. After 4 h of coculture, the cells were washed to remove bacteria and then incubated for 20 h (total of 24 h) in the presence of antibiotics under aerobic conditions (Fig. 1). Next, the phenotype was analyzed by assessing the levels of HLA-ABC, HLA-DR, costimulatory molecules (CD80 and CD86), the maturation marker CD83, and the chemokine receptor CCR7. The DC line without bacterial coculture displayed a characteristic phenotype with easily detectable levels of HLA-ABC, HLA-DR, and CD86 (Figs. 3 and 4) but extremely low levels of CD80, CD83, and CCR7 (Fig. 3). In this study, we assessed the geometric mean fluorescent intensity (MFI) of these molecules on the DC line stimulated with live commensal bacteria and compared them to the unstimulated DC line using Dunnett’s multiple comparisons test. Stimulation of the DC line with live commensal bacteria (*F. varium*, *F. nucleatum*, *B. vulgatus*, *C. clostridioforme*, or *E. coli*) under aerobic or anaerobic conditions resulted in significant upregulation of at least one surface molecule, HLA-ABC, compared to the unstimulated DC line (Figs. 3 and 4, and Supplemental Table 1). On the other hand, *L. bulgaricus* did not upregulate the expression of any molecule on the surface of DCs but significantly decreased HLA-DR levels compared to the unstimulated DC line. Each commensal bacterium exhibited different abilities to modulate the phenotype of the DC line, as shown by the geometric MFIs. Moreover, the increased geometric MFIs of surface molecules on the activated DC line differed between aerobic and anaerobic conditions (Figs. 3 and 4). Interestingly, *F. nucleatum* strongly induced activation of the DC line, as shown by the significant upregulation of all molecules examined under either aerobic or anaerobic conditions, compared to that of the unstimulated DC line (Figs. 3 and 4, and Supplemental Table 1). However, under aerobic or anaerobic conditions, *F. varium* significantly increased only HLA-ABC and HLA-DR expression compared to that of the unstimulated DC line. Coculture of *E. coli* and the DC line under aerobic or anaerobic conditions resulted in significant upregulation of HLA-ABC, CD80, CD86, and CCR7, compared to the unstimulated DC line (Figs. 3 and 4, and Supplemental Table 1). Neither aerobic nor anaerobic cultivation of the DC line stimulated with *B. vulgatus* or *C. clostridioforme* significantly altered the expression of CD83 or CCR7. These results suggested that live commensal bacteria such as *F. nucleatum*, *F. varium*, and *E. coli* activated the DC line at a relatively high level compared to the unstimulated DC line. Moreover, the levels of stimulation of the DC line by the live commensal bacteria used in this study were different under aerobic and anaerobic conditions.

### Changes over time in proinflammatory cytokines produced from the DC line stimulated with live commensal bacteria under aerobic or anaerobic conditions

**Figure 5:**
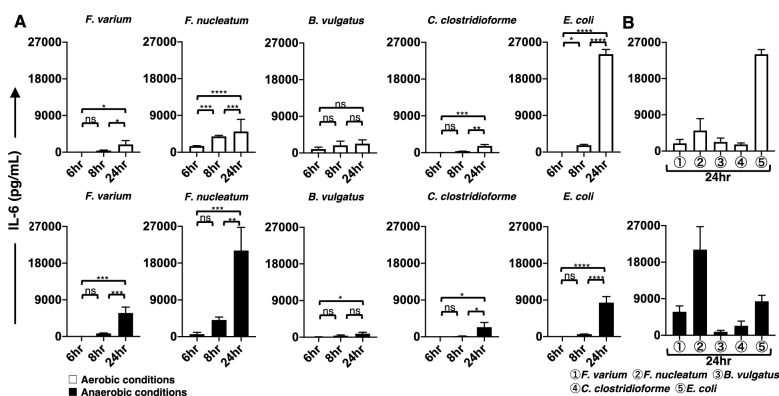
The production of IL-6 by the DC line stimulated with live commensal bacteria. (**A**) Under aerobic (upper panel, white bar) and anaerobic (lower panel, black bar) stimulation conditions, TNF-α production during the initial (total of 6 and 8 h) and late phases (total of 24 h) by the DC line after stimulation with six strains of live commensal bacteria. (**B**) Under aerobic (white bar) and anaerobic (black bar) conditions, IL-6 production during the late phase (total of 24 h) by the DC line after stimulation with six strains of live commensal bacteria was assessed. The six live commensal bacterial strains are as follows: *Fusobacterium varium* (*F. varium*), *Fusobacterium nucleatum* (*F. nucleatum*), *Bacteroides vulgatus* (*B. vulgatus*), *Clostridium clostridioforme* (*C. clostridioforme*), *Escherichia coli* (*E. coli*), and *Lactobacillus delbrueckii subsp. bulgaricus* (*L. bulgaricus*). The results are presented as the means ± SD from three independent experiments. Tukey’s multiple comparisons test was used to compare the levels of IL-6 after 6, 8, and 24 h of culture. ns; not significant, *p < 0.05, **p < 0.01, ***p < 0.001, and ****p < 0.0001.

**Figure 6:**
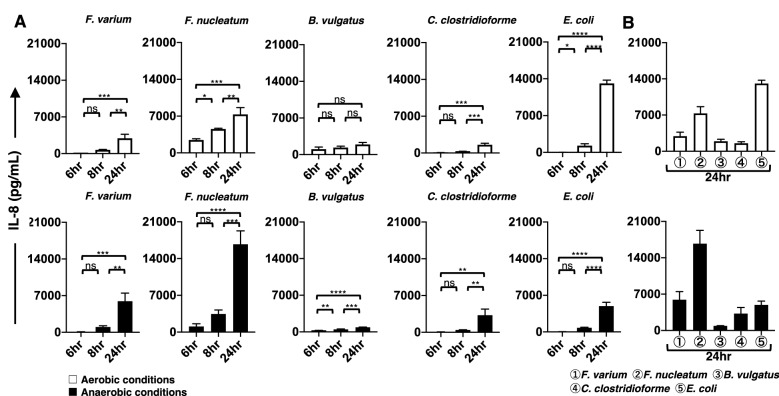
The production of IL-8 by the DC line stimulated with live commensal bacteria. (**A**) Under aerobic (upper panel, white bar) and anaerobic (lower panel, black bar) stimulation conditions, IL-8 production during the initial phase (total of 6 and 8 h) and late phase (a total 24 h) by the DC line after stimulation with six strains of live commensal bacteria was analyzed. (**B**) Under aerobic (white bar) and anaerobic (black bar) stimulation conditions, IL-8 production during the late phase (total of 24 h) by the DC line after stimulation with six strains of live commensal bacteria was analyzed. The six strains of live commensal bacteria are as follows: *Fusobacterium varium* (*F. varium*), *Fusobacterium nucleatum* (*F. nucleatum*), *Bacteroides vulgatus* (*B. vulgatus*), *Clostridium clostridioforme* (*C. clostridioforme*), *Escherichia coli* (*E. coli*), and *Lactobacillus delbrueckii subsp. bulgaricus* (*L. bulgaricus*). The results are presented as the means ± SD from three independent experiments. Tukey’s multiple comparisons test was used to compare the levels of IL-8 after 6, 8, and 24 h of culture. ns; not significant, *p < 0.05, **p < 0.01, ***p < 0.001, and ****p < 0.0001.

**Figure 7:**
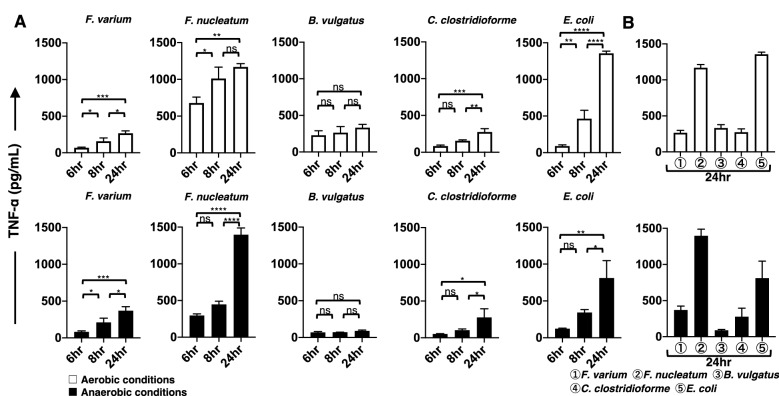
The production of TNF-α by the DC line stimulated with live commensal bacteria. (**A**) Under aerobic (upper panel, white bar) and anaerobic (lower panel, black bar) stimulation conditions, TNF-α production during the initial phase (total of 6 and 8 h) and late phase (total of 24 h) was assessed in the DC line after stimulation with six strains of live commensal bacteria. (**B**) Under aerobic (white bar) and anaerobic (black bar) conditions, TNF-α production during the late phase (total of 24 h) from the DC line after stimulation with six strains of live commensal bacteria was assessed. The six kinds of live commensal bacteria are as follows: *Fusobacterium varium* (*F. varium*), *Fusobacterium nucleatum* (*F. nucleatum*), *Bacteroides vulgatus* (*B. vulgatus*), *Clostridium clostridioforme* (*C. clostridioforme*), *Escherichia coli* (*E. coli*), and *Lactobacillus delbrueckii subsp. bulgaricus* (*L. bulgaricus*). The results are presented as the means ± SD from three independent experiments. Tukey’s multiple comparisons test was used to compare the levels of TNF-α after 6, 8, and 24 h of culture. ns; not significant, *p < 0.05, **p < 0.01, ***p < 0.001, and ****p < 0.0001.

The phenotypic characterization of DCs does not always correlate with functional activation, as assessed by the production of proinflammatory cytokines^29^. Therefore, we next used ELISAs to measure the production of proinflammatory cytokines (IL-4, IL-6, IL-8, IL-10, IL-12p70, interferon (IFN)-γ, and tumor necrosis factor (TNF)-α) by the DC line. In this study, we used both aerobic and anaerobic in vitro models to compare the ability of live commensal bacteria to stimulate cytokine production in the DC line. Cultivation for 4 h under aerobic or anaerobic conditions did not affect the viability of commensal bacteria (*F. varium*, *F. nucleatum*, *B. vulgatus*, *C. clostridioforme*, and *L. bulgaricus*) (Table 1). However, *E. coli.* grew to approximately tenfold higher levels than the other live commensal bacteria after 4 h of culture under either aerobic or anaerobic conditions (Table 1). Therefore, we used approximately 1/10 of the *E. coli* number compared to the other bacteria in this experimental setting. We examined the production of proinflammatory cytokines from the DC line after 4 h of exposure to live commensal bacteria under aerobic or anaerobic conditions. After exposure to live commensal bacteria, the DC line was further cultured under aerobic conditions for 20 h (total of 24 h). The stimulated DC line produced an array of proinflammatory cytokines, such as IL-6 (Fig. 5A), IL-8 (Fig. 6A), and TNF-α (Fig. 7A), under either aerobic or anaerobic conditions. However, IL-4, IL-10, IL-12p70, and IFN-γ production was not detected at any level in the stimulated DC line in this study. Then, the production levels of these cytokines (IL-6, IL-8, and TNF-α) at several culture periods (total of 6, 8, and 24 h) were compared using Tukey’s multiple comparisons test. The production of IL-6, IL-8, or TNF-α by the DC line after exposure to live commensal bacteria, except for *B. vulgatus,* under either aerobic or anaerobic conditions, was significantly increased at 24 h compared to a total of 6 h of culture. However, the production of IL-6 or IL-8 from the DC line after exposure to the anaerobic bacterium *B. vulgatus* resulted in a significant increase at 24 h only under anaerobic conditions compared to a total of 6 h of culture. TNF-α levels produced by the DC line exposed to *B. vulgatus* did not significantly increase in 24 h under either aerobic or anaerobic conditions compared to a total of 6 h of culture. In addition, proinflammatory cytokines were not detected in the cultures of live bacteria alone, the unstimulated DC line, or the *L. bulgaricus*-stimulated DC line in this study (data not shown). These results suggested that different commensal bacteria exhibit different abilities to modulate the proinflammatory responses of the DC line.

### Comparison of the levels of proinflammatory cytokines produced by the DC line upon stimulation with live commensal bacteria

The level of proinflammatory cytokines produced by the DC line at 24 h of culture was compared after stimulation with the different bacteria used in this study (Figs. 5B, 6B, and 7B). According to Tukey’s multiple comparisons test (Supplemental Table 2), the proinflammatory cytokine IL-6, IL-8, and TNF-α levels produced by the DC line stimulated with facultative anaerobes *E. coli* were significantly increased under aerobic conditions compared to those of the DC line stimulated with all other bacteria. However, TNF-α levels produced by the DC line stimulated with *E. coli* under anaerobic stimulation conditions were significantly increased compared to those of cells stimulated with all other bacteria, except for *F. nucleatum*. IL-6 and IL-8 levels derived from the DC line stimulated with *E. coli* were not significantly different from those of cells stimulated with *F. varium* or *C. clostridioforme* under anaerobic but not aerobic stimulation conditions*.* Under anaerobic conditions, the anaerobic bacteria *F. nucleatum*-stimulated DC line produced IL-6, IL-8, and TNF-α at significantly higher levels compared to those of cells stimulated with all other bacteria. However, under aerobic conditions, IL-8 and TNF-α levels produced by the DC line stimulated with *F. nucleatum* were significantly increased compared to those of cells stimulated with all other bacteria, except for *E. coli*. Next, we assessed the production of proinflammatory cytokines by DCs stimulated with *F. varium* or *F. nucleatum* (Supplemental Table 2). Compared to *F. varium*, *F. nucleatum* significantly induced the production of all proinflammatory cytokines in the DC line only under anaerobic conditions. No difference in IL-6 levels was observed between the DC lines stimulated with *F. nucleatum* or *F. varium* under aerobic conditions. The DC line stimulated with *F. nucleatum* produced significantly higher levels of IL-8 or TNF-α than those stimulated with *F. varium* under either aerobic or anaerobic conditions. Although the amount of cytokines produced from the DC line stimulated under aerobic and anaerobic conditions tended to differ between bacterial species, *E. coli, F. nucleatum,* or *F. varium* may be better stimulators of the DC line.

### Comparison of the production of proinflammatory cytokines by the stimulated DC line under aerobic and anaerobic conditions

**Figure 8:**
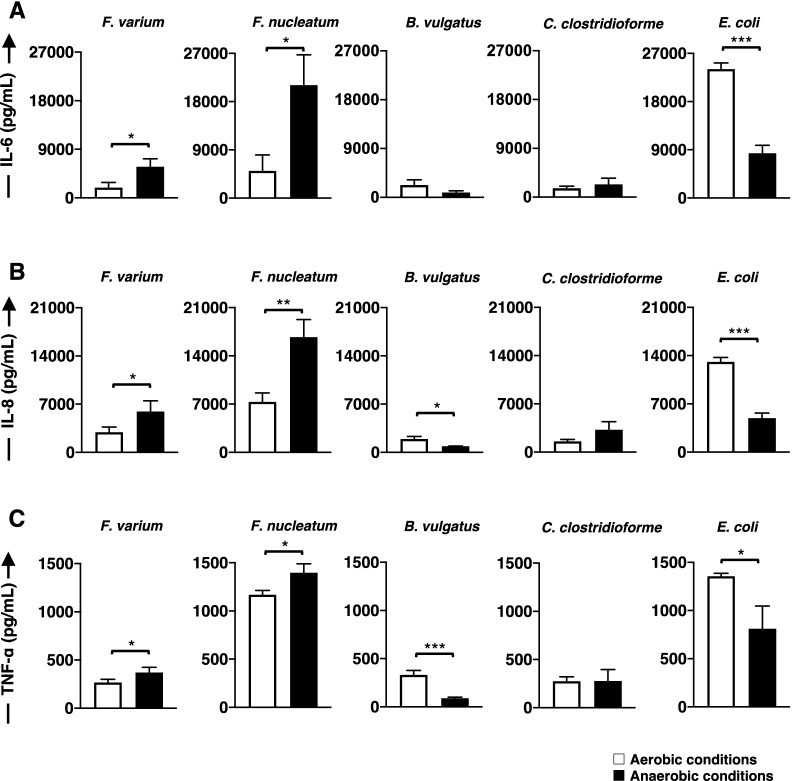
Comparison of the proinflammatory cytokines produced by the DC line stimulated with live commensal bacteria between aerobic and anaerobic conditions. The DC line was stimulated with live commensal bacteria under aerobic (white bar) or anaerobic (black bar) conditions for 4 h. Thereafter, the DC line was cultured for an additional 20 h (total of 24 h) under aerobic culture conditions. IL-6, IL-8, or TNF-α production by the DC line represents the results of three independent experiments. The commensal bacteria used in this study are as follows: *Fusobacterium varium* (*F. varium*), *Fusobacterium nucleatum* (*F. nucleatum*), *Bacteroides vulgatus* (*B. vulgatus*), *Clostridium clostridioforme* (*C. clostridioform*e), and *Escherichia coli* (*E. col*i). A t test was used to compare the levels of proinflammatory cytokines produced by the DC line stimulated with commensal bacteria under aerobic or anaerobic stimulation conditions. *p < 0.05, **p < 0.01, and ***p < 0.001.

Both *F. varium* and *F. nucleatum* significantly induced higher levels of IL-6 (*p* = 0.0194 and 0.0123, respectively), IL-8 (*p* = 0.0405 and 0.0048, respectively), and TNF-α (*p* = 0.0488 and 0.0183, respectively) production by the DC line exposed to anaerobic conditions than aerobic conditions (Fig. 8A–C). Moreover, stimulation of the DC line with the facultative anaerobes *E. coli* under aerobic conditions resulted in significantly higher levels of IL-6 (*p* = 0.0001), IL-8 (*p* = 0.0001), and TNF-α (*p* = 0.0166) production than those under anaerobic conditions (Fig. 8A–C). *B. vulgatus* promoted significantly more IL-8 (*p* = 0.0105) and TNF-α (*p* = 0.0009) production from the DC line under aerobic conditions than under anaerobic conditions, although it is an anaerobic bacterium (Fig. 8B and C). In addition, the DC line stimulated with *C. clostridioforme,* an anaerobic bacterium, produced the same levels of proinflammatory cytokines under aerobic and anaerobic conditions.

## Discussion

In this study, we assessed the effects of commensal bacteria on DC activation by developing an in vitro culture system using a human DC line stimulated with commensal bacteria, including *F. varium*, *F. nucleatum*, *B. vulgatus*, *C. clostridioforme*, and *E. coli*, all of which are pathogens that invade the cytoplasm of colonic epithelial cells^6^. In addition, *L. bulgaricus* was used as a control. We first selected the DC line because it was very difficult to obtain a sufficient number of human MoDCs with extremely high purity. The DC line is easy to establish as an experimental model and T-cell contamination can be avoided. Moreover, the surface phenotype and proinflammatory cytokine production pattern of the DC line are similar to those of MoDCs^19,20^. Therefore, we used the DC line to assess the potential capacity of live commensal bacteria to induce immunomodulation of APCs.

Importantly, the majority of the human gut microbiota comprises anaerobic bacteria that are capable of growing without oxygen. These anaerobic bacteria exhibit essential metabolic functions using various other substances during metabolism in the human colon^30,31^. Because mimicking the exposure of the gut to anaerobic conditions in a human in vitro model is very important, we first developed this model. The DC line used in this study did not survive well without FCS, even for short periods of culture. Moreover, the DC line does not survive and dies after long hours under anaerobic conditions, even in the presence of FCS. Therefore, we first assessed the viability of the DC line exposed to live bacteria without antibiotics under aerobic or anaerobic conditions in the presence of FCS. After 4 h of culture of the DC line with commensal bacteria used in this study under aerobic or anaerobic conditions, the DC line exhibited a viability of more than 75%. Moreover, the viability of the stimulated DC line was not significantly different from that of the unstimulated DC line until 4 h of culture with live commensal bacteria. Therefore, in an in vitro experimental setting, we cocultured the DC line and live commensal anaerobic bacteria (*F. varium*, *F. nucleatum*, *B. vulgatus*, or *C. clostridioforme)* or facultative anaerobes (*L. bulgaricus*) at a 1:100 ratio for 4 h in the presence of FCS but without antibiotics. On the other hand, the facultative anaerobes (*E. coli*) grew to approximately tenfold higher levels than the other live commensal bacteria after 4 h of culture under either aerobic or anaerobic conditions. Therefore, the DC line was cocultured with *E. coli* at a 1:10 ratio. After 4 h of incubation of the DC line with each of the six live commensal bacteria under aerobic or anaerobic conditions, the bacteria were removed, and the fresh medium was replaced with complete medium containing antibiotics to avoid the effect of live bacteria remaining in the culture for an additional 20 h (total 24 h of culture). Then, we assessed the stimulatory effect of the commensal bacteria used in this study on the DC line using this in vitro culture model.

Continuous 4-h stimulation of the DC line with live commensal bacteria under aerobic or anaerobic conditions induced the activation of the DC line with different abilities, as shown by phenotypic analysis. *E. coli* and *F. nucleatum* dramatically differed from other commensal bacteria in their ability to activate the DC line under aerobic or anaerobic conditions, as evidenced by the significant upregulation of almost all surface molecules examined compared to the unstimulated DC line. Although *F. varium* also had the potential to modulate the phenotype of the DC line, *F. varium* did not significantly induce the upregulation of CD83 or CCR7 under either aerobic or anaerobic stimulation conditions compared to the unstimulated DC line*.* In addition, probiotics such as *L. bulgaricus* did not affect the upregulation of the expression of surface molecules on the DC line. The commensal bacteria examined here may have different abilities to activate the immunogenicity of the DC line associated with the pathogenesis of disease caused by complex regulation of immunomodulation by activated DCs. Specifically, *E. coli* and *Fusobacterium* spp. have been suspected to be pathobionts of CRC^21,22^ and IBD^23^. Previous reports indicated that *Fusobacterium* spp., such as *F. nucleatum* and *F. varium,* are gastrointestinal pathogens associated with human diseases, such as CRC^21,31^ and UC^6,12,24^, respectively. Indeed, patients with UC who are treated with antibiotics targeting *F. varium* experience an amelioration of symptoms^32,33^. Moreover, our previous report showed that the commensal microbes used in this study contribute to IBS^20^. The difference in the immunogenicity of DCs after exposure to bacteria may be at least partially associated with the pathogenesis of autoimmune and inflammatory diseases or cancer. However, little is known about how bacteria cause human disease through immunomodulation by DCs.

Phenotypic characterization of DCs does not always correlate with their immunogenicity^29^. Given the different immunomodulatory abilities of live commensal bacteria, we assessed the functional activation of DCs by measuring cytokine production upon stimulation with bacteria under aerobic or anaerobic conditions. In this experimental setting, IL-12p70, IFN-γ, IL-4, and IL-10 were not detected during DC line stimulation with all commensal bacteria under aerobic or anaerobic culture conditions. In this study, we were unable to analyze T-cell responses because autologous T cells derived from the DC line were not available. Therefore, we did not clearly determine whether the DC line stimulated by commensal bacteria induces the differentiation of CD4 + T cells into Th1, Th2, or Th17 cells or Tregs. Mainly, the polarization of these T cells critically depends on the actions of cytokines produced by DC subsets, type-1-polarized DCs producing IL-12p70 and IFN-γ^34^ and type-2-polarized DCs producing IL-4 and IL-10^35^, which induce Th1 and Th2 CD4 + T-cell differentiation, respectively. Interestingly, multifunctional proinflammatory cytokines, such as IL-6, IL-8, and TNF-α, were detected in the DC line after activation by all 5 live commensal bacterial strains examined (*F. varium*, *F. nucleatum*, *B. vulgatus*, *C. clostridioforme*, and *E. coli*) under either aerobic or anaerobic conditions. In addition, no proinflammatory cytokines were detected in unstimulated or probiotic *L. bulgaricus*-stimulated DCs, which also did not express upregulated surface molecules. Our previous study indicated that certain commensal bacteria (*F. varium*, *F. nucleatum*, *B. vulgatus*, *C. clostridioforme*, and *E. coli*)*,* but not probiotics, invade the cytoplasm of colonic epithelial cells, resulting in the production of IL-8 and TNF-α by the cells^6,36^. The mechanism by which these commensal bacteria activate proinflammatory cytokines in the DC line is still unclear. One possibility may be the ability of the DC line to capture these commensal bacteria and become activated^15–17^. Certain commensal bacteria, but not probiotics, may also invade the DC line, activating early intracellular signaling to trigger host inflammatory reactions. The inflammatory responses in the DC line induced by each commensal bacteria showed different levels. Captured commensal bacteria stimulate DCs by ligating TLRs and other pathogen recognition receptors, leading to the expression of costimulatory molecules (CD80 and CD86) and the maturation marker CD83, and the stimulation of antigen-specific T cells^15–17,37^. We and other researchers previously reported that the DC line used in this study expresses TLRs (TLR1, TLR2, TLR4, TLR7 and TLR9), and LPS induces Th1 and Th2 responses through TLR4, as shown by the production of both IL-12p70 and IL-10^16,18–20^ by the DC line. It is difficult to explain without further experiments such as the cultivation of DCs and lymphocytes.

Previous reports indicate that IL-6, IL-8, or TNF-α are produced in excess in patients with IBD or cancer, and increased levels of these cytokines are associated with disease progression^38–42^. Previously, we also documented that certain commensal bacteria used in this study induced inflammation of the epithelium with cryptitis and crypt abscess in patients with UC^6^*.* IL-6 has a very important role in regulating the balance between Th17 CD4 + T cells and Tregs^40^. In this situation, IL-6 induces the development of IL-17-producing Th17 CD4 + T cells from naive T cells together with TGF-β and inhibits Treg differentiation induced by TGF-β^40^. Th17 CD4 + T cells are involved in the pathogenesis of IBD and pathologic inflammatory states^40^. On the other hand, IL-8 polymorphisms are associated with altered susceptibility to IBD or cancer^43^. Th17 CD4 + T cells produce a number of proinflammatory cytokines, such as IL-17, IL-21, IL-22, and TNF-α, and play a key role in mucosal defense against various pathogens^44^. IL-17, IL-21, IL-22, TNF-α and IL-6 are also produced in excess in individuals with CRC and synergistically promote tumor growth^42–44^. DCs stimulated by certain commensal bacteria may mediate CD4 + T-cell differentiation into Th1, Th2, or Th17 cells, all of which alter gut immune homeostasis and lead to IBD or cancer. IL-6, IL-8, or TNF-α production by DCs stimulated by commensal bacteria, demonstrated in this study, may be associated with IBD and cancer. In the clinic, anti-cytokine agents have been proposed to be useful for inhibiting inflammation in IBD and autoimmune diseases, such as rheumatoid arthritis, resulting in long-term control of inflammation^39^.

As anaerobic culture conditions mimic intestinal environments, aerobic conditions are disadvantageous for their survival. In this study, we compared the production of proinflammatory cytokines by the DC line between aerobic and anaerobic conditions using this in vitro culture model. In the present study, we used anaerobic bacteria (*F. varium*, *F. nucleatum*, *B. vulgatus*, and *C. clostridioforme*) and facultative anaerobes (*E. coli*). High levels of IL-6, IL-8, or TNF-α were produced by the DC line stimulated with *E. coli* under both aerobic and anaerobic conditions. Both aerobic and anaerobic conditions may not be disadvantageous for *E. coli*. However, significantly higher IL-6, IL-8, or TNF-α levels were produced by the DC line stimulated with *E. coli* under aerobic conditions than under anaerobic conditions. Therefore, the *E. coli* strain used in this study may be capable of significantly stimulating the DC line under aerobic conditions. Presumably, this trend varies depending on the strain used. Fusobacteria (*F. varium* and *F. nucleatum*) stimulated the DC line to produce significantly higher IL-6, IL-8, or TNF-α levels under anaerobic conditions than under aerobic conditions. Therefore, anaerobic bacteria, such as *F. varium* and *F. nucleatum*, are better stimulators of the DC line under anaerobic conditions that mimic intestinal environments. However, an anaerobic bacterium, *B. vulgatus,* promoted significantly more IL-8 and TNF-α production by the DC line under aerobic conditions than under anaerobic conditions. Since the number of anaerobic bacteria did not change significantly after 4 h of aerobic stimulation, the production of proinflammatory cytokines is unlikely to involve dead bacteria. The ability of live anaerobic bacteria to stimulate the DC line under aerobic or anaerobic conditions may differ. The effect of fusobacteria on the DC line should be evaluated under anaerobic conditions similar to the colonic environment, since fusobacteria stimulation significantly increased the production of proinflammatory cytokines by the DC line under anaerobic conditions.

We further discussed the effects of *F. varium* and *F. nucleatum* on the DC line under anaerobic conditions. Compared to *F. varium*, *F. nucleatum* stimulated the DC line, as evidenced by the upregulation of surface molecules on the DC line and production of IL-6, IL-8, or TNF-α at significantly higher levels. Previous studies have indicated an association between *F. nucleatum* and the progression of advanced CRC^21,32^. Components of the human gut microbiota, such as *F. nucleatum,* may contribute to the etiology of advanced CRC, not only via the procarcinogenic activities of *F. nucleatum* but also via the effect of wider microbe-induced proinflammation^45^. Furthermore, *F. varium* is one of the pathogens causing UC^6,12,24^*.* The *F. varium-*stimulated DC line produced high levels of IL-6, IL-8, or TNF-α, consistent with our previous report indicating that *F. varium* in actively inflamed colonic mucosa was associated with the progression of UC^36^ and the pathogenesis of colorectal adenoma and intramucosal CRC^25^. *E. coli, F. nucleatum,* and *F. varium,* which induce the production of high levels of proinflammatory cytokines from activated DCs, may be potential pathogens causing various inflammatory diseases or cancers.

Although the intestinal tract is an anaerobic environment and most intestinal bacteria are anaerobic bacteria, crosstalk between intestinal bacteria and immune cells, including DCs, has previously been evaluated under aerobic conditions. This study is significant as the first paper to analyze this process under anaerobic conditions. Under anaerobic conditions similar to intestinal environments, *E. coli*, *F. nucleatum, and F. varium* were the stimulatory commensal bacteria affecting the DC line. The next step of the research is to analyze the effects of commensal anaerobic bacteria on autologous immune-related cells, including DCs and T cells, from patients with IBD or CRC. Identification of the mechanisms by which anaerobic bacteria affect the patient's immune system and cause disease is important.

## Methods

### Cells and conditioned medium

The human DC line (PMDC05) has myeloid activity in the human DC lineage and functions as an APC to induce immunomodulation^16,18^. This line was a kind gift from Dr. Takahashi (Laboratory of Hematology and Oncology, Graduate School of Health Sciences, Niigata University, Niigata, Japan). The DC line was maintained in Iscove's modified Dulbecco's medium (IMDM) (Sigma–Aldrich; Merck KGaA, Darmstadt, Germany) supplemented with 100 U/mL penicillin, 100 μg/mL streptomycin (Wako Pure Chemical Industries, Ltd., Osaka, Japan) and 10% fetal calf serum (FCS) (Cytiva, Marlborough, MA, United States) under aerobic conditions in a humidified CO_2_ incubator (5% CO_2_ at 37 °C).

### Preparation of commensal bacteria

Human commensal bacteria were obtained from the American Type Culture Collection (ATCC, Rockville, MD, United States) or Japan Collection of Microorganisms (JCM, RIKEN, Wako, Japan): *F. varium* (ATCC8501), *F. nucleatum* (ATCC25586), *B vulgatus* (JCM5826), *C. clostridioforme* (JCM1219), and *E. coli* (JCM1649). As these bacteria invade colonic epithelial cells and activate early intracellular signaling pathways to trigger host inflammation^6^, we selected these bacteria in the present study. Moreover, a probiotic, *L. bulgaricus* (LB-021001; Meiji Dairies), was used as a control. *E. coli* was harvested from BTB agar plates (Eiken Chemical Co. Ltd., Tokyo, Japan). *B. vulgatus* was harvested from Bacteroides agar plates (Nissui Chemical Co. Ltd., Tokyo, Japan). *C. clostridioforme* and *L. bulgaricus* were harvested from ABCM agar plates (Eiken Chemical Co. Ltd.). *F. nucleatum* and *F. varium* were harvested from FM agar-modified plates (Nissui Chemical Co. Ltd., Tokyo, Japan). These plates were cultured at 37 ℃ for 24–48 h. Facultative anaerobes (*E. coli*) were incubated under aerobic conditions in a humidified CO_2_ incubator (5% CO_2_ at 37 °C). Anaerobic bacteria (*F. varium*, *F. nucleatum*, *B. vulgatus*, and *C. clostridioforme*) and facultative anaerobes (*L. bulgaricus*) were incubated in a gas generator for anaerobic culture using Anaeropack Kenki (Mitsubishi Gas Chemical Co., Inc., Tokyo, Japan) (< 0.1% O_2_, > 16% CO_2_ at 37 °C). After culture, the colonies were collected using a disposable plastic loop and suspended at a density of 1 × 10^8^ colony forming units (CFUs)/mL in IMDM without antibiotics or FCS.

### Stimulation of the DC line with live commensal bacteria

The human DC line (1 × 10^6^ cells/mL) was incubated with each live commensal bacterial strain (1 × 10^8^/mL) (*F. varium*, *F. nucleatum*, *B. vulgatus*, *C. clostridioforme*, *L. bulgaricus*, or *E. coli* (JCM1649) (1 × 10^7^/mL) in 1 mL of IMDM supplemented with 10% FCS in the absence of antibiotics in a 24-well plate for 4 h under aerobic (5% CO_2_ at 37 °C) or anaerobic (< 0.1% O_2_, > 16% CO_2_ at 37 °C) culture conditions. After coincubation, the cells were washed three times with PBS (Wako Pure Chemical Industries, Ltd.) to remove commensal bacteria and further incubated with IMDM with 10% FCS in the presence of 100 U/mL penicillin and 100 μg/mL streptomycin for an additional 2, 4, or 20 h under aerobic conditions (5% CO_2_ at 37 °C) (Fig. 1).

### Viability of the DC line stimulated with live commensal bacteria

Trypan blue solution (Sigma-Aldrich; Merck KGaA) was used to assess the viability of the DC line after coculture with live commensal bacteria according to the manufacturer's protocols. Cell viability was assessed using a CKX41 light microscope (magnification, × 100; Olympus Corporation).

### The change in the commensal bacteria number after culture

The commensal bacteria used in this study were suspended in IMDM supplemented with 10% FCS without antibiotics, and the concentration was adjusted to 1 × 10^8^ CFUs/mL using a turbidimeter (HACH 2100 N, DKK-TOA Corporation, Tokyo, Japan). Then, the commensal bacteria were cultured for 4 h under aerobic or anaerobic conditions, and the viable bacteria were counted. The changes in viable bacterial counts were evaluated logarithmically. For this experiment, increases were evaluated as 10 times or higher bacterial counts (log10 (CFUs/mL)), and decreases were evaluated as 1/10 or less bacterial counts (log10 (CFUs/mL)). When none of the aforementioned logarithmic changes were observed, the bacterial number was considered unchanged.

### Phenotypic analysis

The DC line was cocultured with each live commensal bacteria for 4 h under aerobic (5% CO_2_ at 37 °C) or anaerobic culture conditions (< 0.1% O_2_, > 16% CO_2_ at 37 °C) to assess the cellular phenotype. After coincubation, the cells were washed three times with PBS to remove the commensal bacteria and subsequently incubated with IMDM supplemented with 10% FCS in the presence of 100 U/mL penicillin and 100 μg/mL streptomycin for 20 h under aerobic (5% CO_2_ at 37 °C) culture conditions (Fig. 1). Moreover, the unstimulated DC line was used as a control. Cells were washed three times with PBS and incubated with the following monoclonal antibodies (mAbs): fluorescein isothiocyanate (FITC)-conjugated anti-human HLA-ABC (W6/32), CD80 (2D10) (all from BioLegend, San Diego, CA, United States), phycoerythrin (PE)-conjugated anti-human HLA-DR (L243), CD83 (HB 15e), CD86 (IT2.2) (all from BioLegend), and CCR7 (150,503, R&D Systems, Minneapolis, MN, United States). The DC phenotype was analyzed using FlowJo analysis software (Tree Star, Ashland, OR, United States). The geometric MFI of the indicated molecules, which were expressed by the derived DC line, was analyzed by subtracting the background MFI of unstained controls from the geometric MFI of each molecule. The results are reported as the means ± SD of three independent experiments.

### Enzyme-linked immunosorbent assay (ELISA)

We examined whether the human DC line produced cytokines (IL-4, IL-6, IL-8, IL-10, IL-12p70, IFN-γ and TNF-α) upon stimulation with six strains of live commensal bacteria under aerobic or anaerobic conditions for 4 h. The cells were washed three times with PBS to remove commensal bacteria and further incubated with IMDM supplemented with 10% FCS in the presence of 100 U/mL penicillin and 100 μg/mL streptomycin for 2, 4 or 20 h under aerobic (5% CO_2_ at 37 °C) culture conditions. At three time points, supernatants were collected from each sample and tested using ELISAs (R&D Systems, Inc.) according to the manufacturer’s instructions. Background cytokine levels in conditioned medium were subtracted from each sample. As the viability of the DC line was affected in the culture of the DC line with bacteria in aerobic and anaerobic conditions, implying the different number of live cytokine-producing cells in different cultures, the concentration of the measured cytokines should be corrected with the factor of DC viability. Therefore, cytokine production was corrected using the following formula based on the viability of the unstimulated DC line: cytokine production after correction = (cytokine production before correction) × (viability of the unstimulated DC line)/(viability of the stimulated DC line). Data represent the results from three independent experiments. The results are presented as the means ± SD.

### Statistical analysis

One-way analysis of variance (ANOVA) and Tukey’s multiple comparisons test were used for ELISAs, since we were analyzing multiple groups in these experiments. Moreover, one-way ANOVA and Dunnett`s multiple comparisons test were used to analyze cell viability or the geometric MFI by comparing the mean value for every sample to the mean value of the control group. Differences in the production of proinflammatory cytokines by the DC line cultured under aerobic or anaerobic conditions were evaluated using the t test. *p* < 0.05 was considered to indicate a statistically significant difference. The statistical analyses were conducted using GraphPad Prism version 8.4.3 software (GraphPad Software, Inc., San Diego, CA).

## Supplementary Information

Supplementary Information 1.

Supplementary Information 2.

## Data Availability

All data generated or analyzed during this study are included in this published article [and its supplementary information files].
